# Operando ESR observation in thermally activated delayed fluorescent organic light-emitting diodes

**DOI:** 10.1038/s41598-023-38063-3

**Published:** 2023-07-10

**Authors:** Shintaro Yumoto, Junya Katsumata, Fumiya Osawa, Yoshimasa Wada, Katsuaki Suzuki, Hironori Kaji, Kazuhiro Marumoto

**Affiliations:** 1 Department of Materials Science, Institute of Pure and Applied Sciences, University of Tsukuba, Tsukuba, Ibaraki 305-8573 Japan; 2 Institute for Chemical Research, Kyoto University, Uji, Kyoto 611-0011 Japan; 3 Tsukuba Research Center for Energy Materials Science (TREMS), University of Tsukuba, Ibaraki, 305-8571 Japan

**Keywords:** Organic LEDs, Electronic devices, Organic LEDs

## Abstract

Organic light-emitting diodes (OLEDs) using thermally activated delayed fluorescence (TADF) materials have advantages over OLEDs using conventional fluorescent materials or high-cost phosphorescent materials, including higher efficiency and lower cost. To attain further high device performance, clarifying internal charge states in OLEDs at a microscopic viewpoint is crucial; however, only a few such studies have been performed. Here, we report a microscopic investigation into internal charge states in OLEDs with a TADF material by electron spin resonance (ESR) at a molecular level. We observed operando ESR signals of the OLEDs and identified their origins due to a hole-transport material PEDOT:PSS, gap states at an electron-injection layer, and a host material CBP in the light-emitting layer by performing density functional theory calculation and studying thin films used in the OLEDs. The ESR intensity varied with increasing applied bias before and after the light emission. We find leakage electrons in the OLED at a molecular level, which is suppressed by a further electron-blocking layer MoO_3_ between the PEDOT:PSS and light-emitting layer, resulting in the enhancement of luminance with a low-voltage drive. Such microscopic information and applying our method to other OLEDs will further improve the OLED performance from the microscopic viewpoint.

## Introduction

Organic light-emitting diodes (OLEDs) have characteristic features such as flexibility, light weight, and highly efficient electroluminescence^1–7^. Thus, they have been attracting high attention because of the application to displays such as smartphones, televisions, etc^1–7^. In 1987, Tang and VanSlyke have developed fluorescence OLEDs with tris(8-hydroxyquinolinato)aluminum (Alq_3_) as a luminescent material^1^. After that, highly efficient OLEDs using phosphorescent materials have shown that they have nearly 100% internal quantum efficiency^8,9^. However, blue phosphorescence OLEDs still have problems such as short lifetimes, the use of rare metals (Ir, Pt, etc.) for environmental issues, etc^10^. In 2009, Adachi et al. have developed OLEDs with thermally activated delayed fluorescence (TADF) as a new light-emitting mechanism^11–16^. TADF has an emission mechanism with not only conventional fluorescence from excited singlet states but also delayed fluorescence due to reverse intersystem crossing (RISC) from excited triplet states to excited singlet states^13^. This mechanism shows a high luminescence efficiency that is almost as same as that of phosphorescent OLEDs^17–20^.

Early OLEDs were often fabricated with a vapor deposition method, but in recent years, OLEDs have been fabricated with a solution process, which is expected to contribute to low-cost and mass production^21^. However, there are still rooms for clarifying the problems such as a roll-off of luminance efficiency by applying high voltages, driving lifetimes, and an overproduction of excitons by a small rate constant of RISC in TADF materials^22–26^. Clarifications by observations of electric charge states, electron orbitals, etc. at the molecular level are necessary under device operation. Although there are a lot of studies about materials and molecular design, there are few research at the microscopic point of view during the operation of OLEDs with TADF materials. Thus, it is important to directly observe the charge states in OLEDs with TADF materials from the microscopic point of view.

One of the most suitable techniques to study electric charge states in OLEDs is electron spin resonance (ESR) spectroscopy^27,28^. The ESR method is a highly sensitive and nondestructive technique for directly observing radicals and charges with spins in semiconductors and their devices at the molecular level. So far, we have been studying microscopic nature such as electric charge states, electron spin states, and molecular orientations in organic devices, etc. using the ESR method^28–39^. We have reported an ESR study of OLEDs with Alq_3_ to investigate the degradation mechanism, which have shown the correlation between luminescence degradation and formation of cationic species due to decomposed Alq_3_ molecules^28^. However, an operation mechanism of OLEDs has not yet been investigated using ESR under device operation from a microscopic viewpoint at a molecular level. Moreover, OLEDs with TADF materials, which show high efficiency compared to that of OLEDs with Alq_3_, have not yet been investigated with the ESR method. Such microscopic investigation of OLEDs with TADF materials will be useful for the development of even more efficient OLEDs.

**Figure 1:**
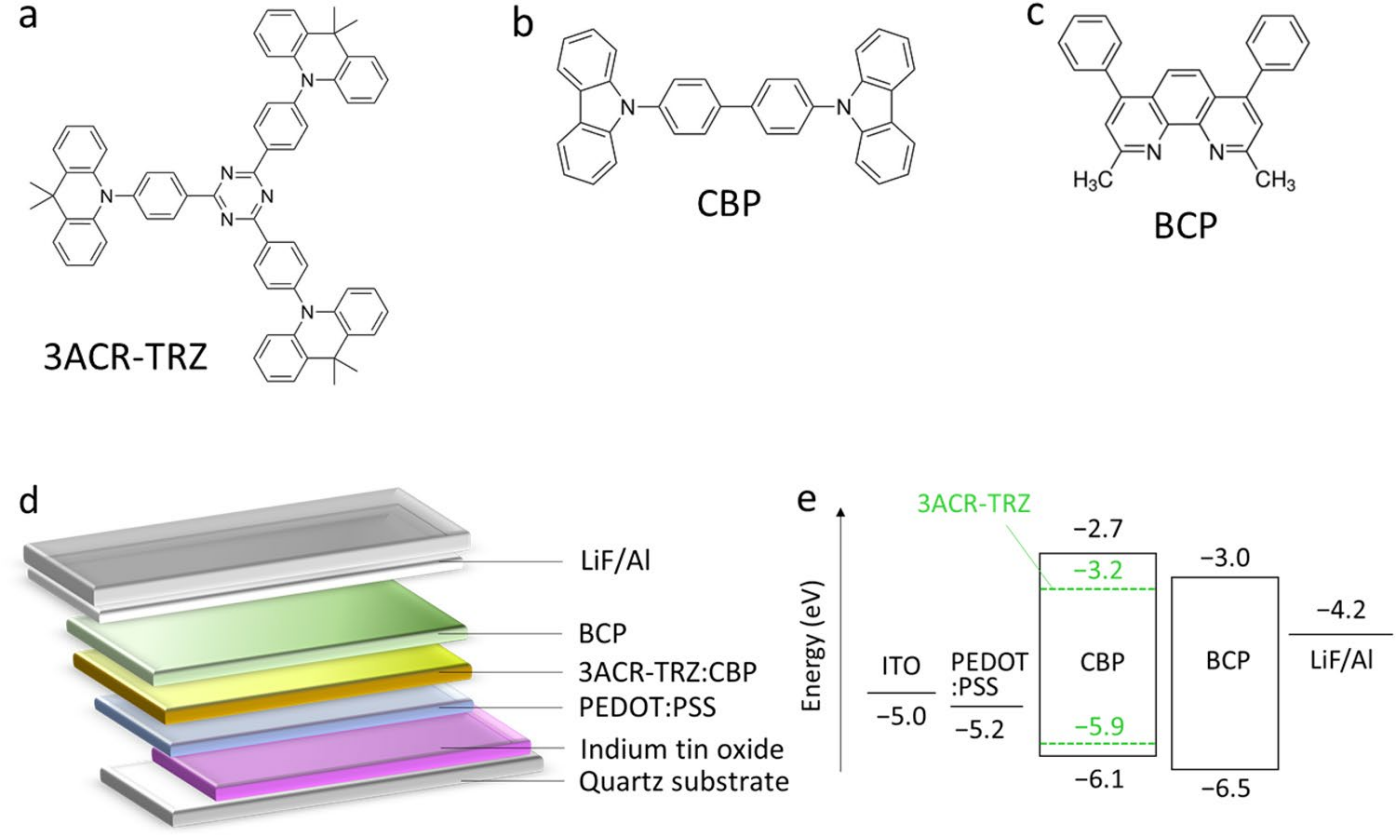
Materials and device structure of OLED with TADF. (**a**–**c**) Chemical structures of (**a**) a TADF material 3ACR-TRZ, (**b**) a host material CBP in the light-emitting layer, and (**c**) an electron transport layer (a hole blocking layer) bathocuproine (BCP). (**d**) A schematic of the OLED structure. (**e**) Energy level diagram of the materials used for the OLED.

Here, we report microscopic investigation of OLEDs with a TADF material under device operation (operando states) for the first time. We utilize 2,4,6-tris(4-9,9-dimethylacridan-10-yl)phenyl)-1,3,5-triazine (3ACR-TRZ)^21^ as a TADF guest material in the light-emitting layer (Fig. 1a). We study the electric charge states in OLEDs using an operando ESR method in detail. As a result, we firstly observe operando ESR signals of the OLEDs with the TADF material. We analyze the origins of the ESR signals using density functional theory calculation and clarify the dependence of the ESR intensity on applied voltage bias (*V*_bias_) to the OLEDs. We find de-doping process of a hole transport layer and long-lived doping states in the light-emitting layer, which are useful information for understanding the operation mechanism of OLEDs with higher efficiency. These findings will be useful for fabricating and developing highly efficient and durable devices.

## Results and discussion

### OLED device structures and characterization methods

To attain a high signal-to-noise ratio (S/N ratio) of the ESR signal by increasing the light-emitting area of the devices, we utilized a rectangular device structure (3 mm × 25 mm) in an ESR sample tube with an inner diameter of 3.5 mm^33,36^. Figure 1a–c shows the chemical structures of molecules used for the device fabrication. Figure 1d shows the fabricated device structure, indium tin oxide (ITO) (150 nm)/poly(3,4-ethylenedioxythiophene):poly(styrene sulfonate) (PEDOT:PSS) (35 nm)/3ACR-TRZ(16 wt%):CBP (55 nm)/bathocuproine (BCP) (30 nm)/lithium fluoride (LiF) (1 nm)/Al (80 nm). Figure 1e shows the energy level diagram of the materials used for the OLED. The energy values of the materials except for BCP are taken from Ref.^21^ and those of BCP are taken from Ref.^40^. A film of PEDOT:PSS as a hole transport layer and a blend film of 3ACR-TRZ:CBP as a light-emitting layer were deposited on an ITO substrate with a spin-coating method. BCP, LiF, and Al were vapor-deposited sequentially on the light-emitting layer. Our device architecture is a typical one and it is important to study the typical device architecture using PEDOT:PSS, BCP, etc. because there are many examples of research on typical device architectures and their problems should be clarified^11–21,40^. The device for ESR and current density (*J*)-luminance measurements was sealed in an ESR sample tube with a desiccant and sealant under a nitrogen glove-box atmosphere^28,36^. Also, to identify the ESR signals’ origins, we fabricated thin-film samples of BCP/LiF/Al and PEDOT:PSS on nonmagnetic quartz substrates with the same fabrication method used for the OLED fabrication. The fabricated OLEDs were simultaneously measured with an ESR spectrometer (JEOL JES-FA200, X-Band 9.5 GHz) and a luminance meter (TOPCON BM-9 M) at room temperature. We used a lock-in detection method for the ESR measurements with a modulation frequency of 100 kHz for the external magnetic field. Thus, we could observe long-lived charges (spins) with a lifetime of > 10 μs or accumulated charges in the OLEDs, and cannot observe charges with a lifetime of < 10 μs that contribute the standard operation of the OLEDs^28–31,36–39,41^. We measured operando ESR signals while increasing *V*_bias_ from 0 to 10 V in 0.5 V increment. A standard Mn^2+^ marker sample was employed as a standard sample to calibrate the peak-to-peak ESR linewidth (Δ*H*_pp_) and the number of spins (*N*_spin_) of observed ESR spectra. The *N*_spin_ was evaluated by integrating the ESR spectrum twice and by comparing the Mn^2+^ standard sample. More detailed fabrication methods and ESR evaluation method are described in “Methods” section.

### Operando ESR spectra of TADF OLEDs and their molecular origins

**Figure 2:**
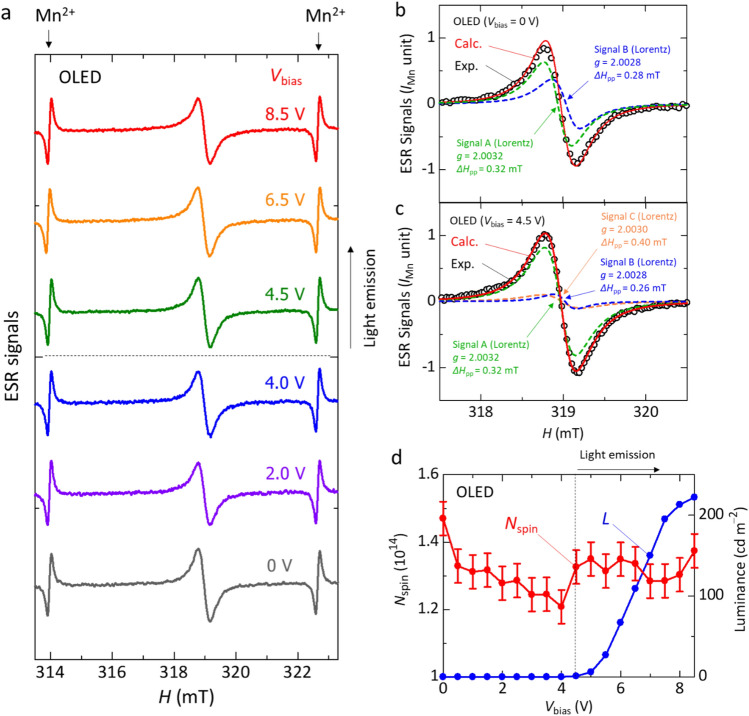
Operando ESR study of the TADF OLED. (**a**) *V*_bias_ dependence of the operando ESR spectra of the OLED. (**b**,**c**) Fitting analyses of the ESR spectra of the OLED before device operation at *V*_bias_ = 0 V (**b**) and after light emission at *V*_bias_ = 4.5 V (**c**). (**d**) Dependence of the number of spins (*N*_spin_) derived from ESR spectra of the OLED and the luminance (*L*) of the OLED on *V*_bias_ during device operation.

We present the observation of operando ESR signals under device operation and compare them with simultaneously measured *J* and luminance *L* of the OLED as a function of *V*_bias_. Figure 2a shows the *V*_bias_ dependence of the operando ESR spectra of the OLED. The electroluminescence characteristics of our fabricated OLEDs are as follows. When we fabricated devices using a conventional ITO substrate (25 mm × 25 mm), we obtained electroluminescence characteristics as shown in Supplementary Fig. S1. The light emission started at a turn-on voltage of *V*_bias_ = 4.2 V and the luminance reached 2200 cd m^−2^ at *V*_bias_ = 7.4 V. Here, the turn-on voltage is defined as the threshold luminance level of 1 cd m^−2^. The maximum current efficiency was obtained as ~ 4.5 cd A^−1^ at *V*_bias_ = 7.1 V, which was evaluated from the data shown in Supplementary Fig. S1a. The OLED emitted green light with at a peak wavelength of 523 nm due to the TADF material 3ACR-TRZ as shown in Supplementary Fig. S1b, which is consistent with that of the previous study^21^. When we fabricated devices for ESR measurements using a rectangular ITO substrate (3 mm × 25 mm, see Fig. 1d), the OLED emitted green light due to 3ACR-TRZ as shown in Supplementary Fig. S2, which is like that observed for the OLEDs fabricated with the conventional ITO substrate (25 mm × 25 mm) mentioned above. The light emission started at a turn-on voltage of *V*_bias_ = 4.5 V (Fig. 2d). The luminance reached 237 cd m^−2^ at *V*_bias_ = 9.5 V, and the maximum current efficiency was obtained as ~ 2.3 cd A^−1^ at *V*_bias_ = 6.5 V (Fig. 1d). The reason for the lower device performance obtained with the devices used for the ESR measurements may be due to the lower film quality compared to the conventional substrate used.

We observed two variations in the ESR spectra before the light emission (0–4.0 V) and after the light emission (*V*_bias_ ≥ 4.5 V); the origins of the ESR spectra are due to long-lived charges (spins) with a lifetime of > 10 μs or accumulated charges in the OLEDs, not due to charges with a lifetime of < 10 μs that contribute the standard operation of the OLEDs, as mentioned above^28–31,36–39,41^. We have found that the observed ESR spectra show no anisotropy, which demonstrates that the charge-accumulation sites prove to be amorphous. We obtained the ESR parameters, *g*-factor and peak-to-peak ESR linewidth (Δ*H*_pp_), as *g* = 2.0029 ± 0.0001 and Δ*H*_pp_ = 0.38 ± 0.02 mT at *V*_bias_ = 0 V, and *g* = 2.0029 ± 0.0001 and Δ*H*_pp_ = 0.40 ± 0.02 mT at *V*_bias_ = 4.5 V, which are summarized in Supplementary Table S1.

In general, an ESR spectrum due to charges with unpaired electrons in semiconductors can be described with a Lorentzian function or a Gaussian function; while the Lorentzian function describes the states where charges are mobile, the Gaussian function describes the states where charges are immobile^28–39^. Since the observed ESR spectra cannot be described by a single component, we performed fitting analyses of the ESR spectra with several components using a least-squares method and two kinds of software of Igor Pro (Ver. 6.36J) and EasySpin (Ver. 5.2.18). We utilized the following Equations for the fitting analysis:

1 $${I}_{\mathrm{L}}\left(\mathrm{H}\right)= -\frac{2{h}_{\mathrm{L}}\left(H-{H}_{0,\mathrm{L}}\right)}{{{w}_{\mathrm{L}}}^{2}{\left[1+{\left(\frac{H-{H}_{0,\mathrm{L}}}{{w}_{\mathrm{L}}}\right)}^{2}\right]}^{2}}$$

2 $${I}_{\mathrm{G}}\left(\mathrm{H}\right)= -2\frac{2{h}_{\mathrm{G}}\left(H-{H}_{0,\mathrm{L}}\right)}{{{w}_{\mathrm{G}}}^{2}}\mathrm{exp}\left({-\left(\frac{H-{H}_{0,\mathrm{G}}}{{w}_{\mathrm{G}}}\right)}^{2}\right)$$

Equations (1) and (2) describes the Lorentzian and Gaussian functions, respectively. Here, we used a resonance magnetic field $({H}_{0,\mathrm{L}}, {H}_{0,\mathrm{G}})$, a half width at half maximum $\left({w}_{\mathrm{L}}, {w}_{\mathrm{G}}\right)$, and a spectrum height $({h}_{\mathrm{L}}, {h}_{\mathrm{G}})$ as fitting parameters, where the subscript “L” and “G” indicate the parameters for the Lorentzian and Gaussian functions, respectively.

We start to analyze the ESR spectrum of the OLED before device operation (*V*_bias_ = 0 V). After examining the combinations of Eqs. (1) and (2), we have found the fitting curve with two components of the Lorentzian function reproduces the ESR spectrum at *V*_bias_ = 0 V. Here, the equation we used is shown below:

3 $$I\left(H\right)= {I}_{\mathrm{L}1}\left(H\right)+{I}_{\mathrm{L}2}\left(H\right)= -\left\{\frac{2{h}_{\mathrm{L}1}(H-{H}_{0,\mathrm{L}1})}{{{w}_{\mathrm{L}1}}^{2}{\left[1+{\left(\frac{H-{H}_{0,\mathrm{L}1}}{{w}_{\mathrm{L}1}}\right)}^{2}\right]}^{2}}+\frac{2{h}_{\mathrm{L}2}(H-{H}_{0,\mathrm{L}2})}{{{w}_{\mathrm{L}2}}^{2}{\left[1+{\left(\frac{H-{H}_{0,\mathrm{L}2}}{{w}_{\mathrm{L}2}}\right)}^{2}\right]}^{2}}\right\}$$

Figure 2b shows the fitting result of the ESR spectrum before device operation (*V*_bias_ = 0 V). Black circles in Fig. 2b show the experimental data of the ESR spectrum. A red line shows the fitting result composed of Signal A (green dotted line) and Signal B (blue dotted line). As discussed later, the origins of Signal A and Signal B are mobile charges with spins, in which case the ESR signal is well described by a Lorentzian lineshape. We obtained the ESR parameters, *g*-factor and Δ*H*_pp_, from the fitting result. The ESR parameters of Signal A are *g* = 2.0032 ± 0.0001 and Δ*H*_pp_ = 0.32 ± 0.04 mT, and those of Signal B were *g* = 2.0028 ± 0.0001 and Δ*H*_pp_ = 0.28 ± 0.04 mT. The parameters obtained from the fitting analysis are summarized in Supplementary Table S1. We have confirmed that the ESR spectra before the light emission (0 V ≤ *V*_bias_ ≤ 4.0 V) are reproduced with Signal A and Signal B. The *g*-factor is calculated using the magnetic resonance equation, $h\nu =g{\mu }_{\mathrm{B}}{H}_{0}$. Here, *h* is the Planck constant, *ν* is the microwave frequency in this study, ${\mu }_{\mathrm{B}}$ is Bohr magneton, and ${H}_{0}$ is the resonance magnetic field where the ESR spectrum with first derivative form has a value of zero. The Δ*H*_pp_ value is calculated from the fitting parameters as $\Delta {H}_{\mathrm{pp}}=(2/\sqrt{3}){w}_{\mathrm{L}}$ in the Lorentz function and $\Delta {H}_{\mathrm{pp}}=\sqrt{2}{w}_{\mathrm{G}}$ in the Gauss function, which means the difference between the two magnetic fields at a peak and valley in the ESR spectrum.

**Figure 3:**
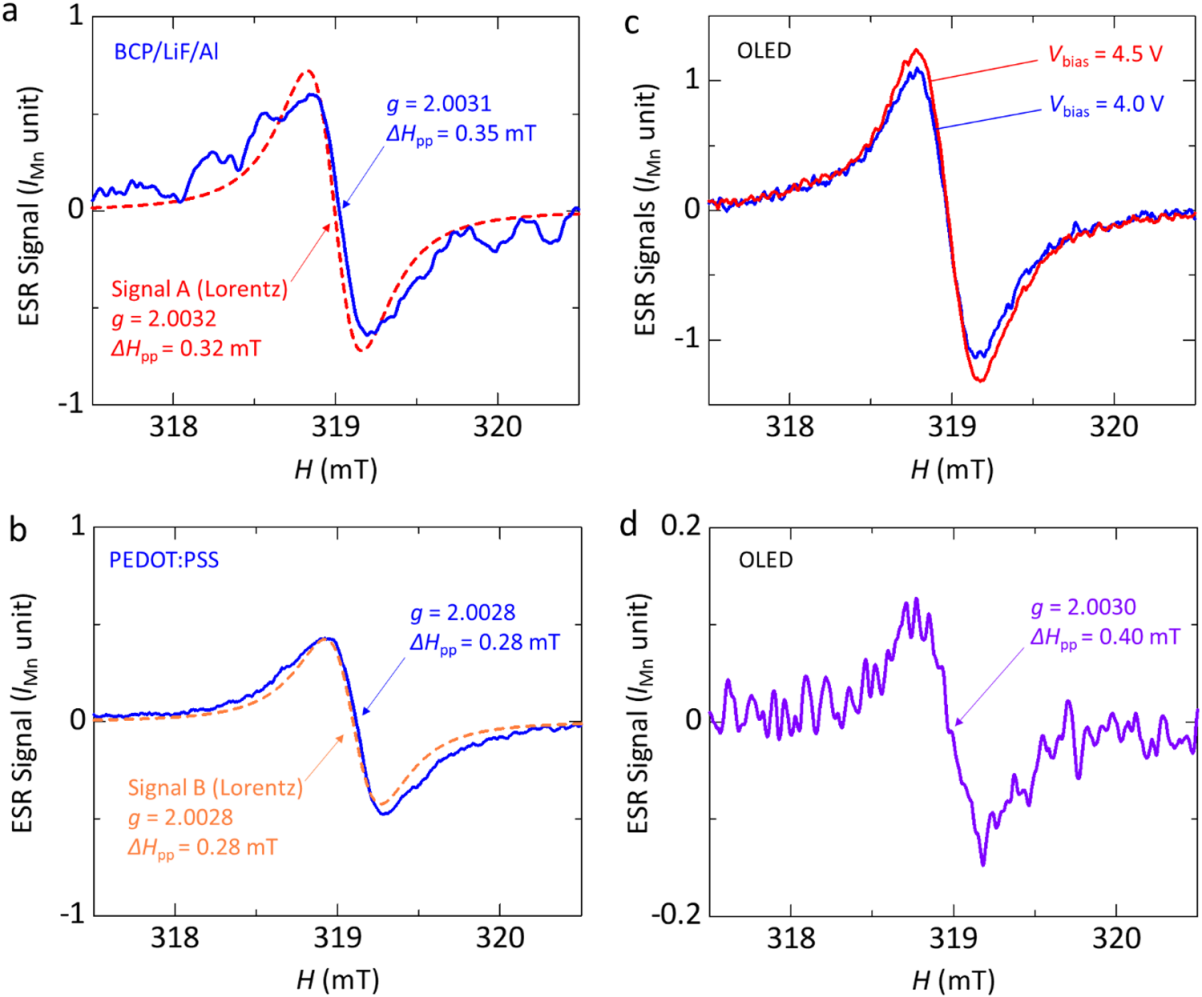
ESR spectra of layered films and ESR variation of the OLED. (**a**) ESR spectrum of a BCP/LiF/Al layered film. (**b**) ESR spectrum of a PEDOT:PSS layered film. (**c**) ESR spectra of the OLED before light emission at *V*_bias_ = 4.0 V and after light emission at *V*_bias_ = 4.5 V. (**d**) Difference in ESR spectra at *V*_bias_ = 4.5 V and 4.0 V.

The origins of Signal A and Signal B obtained from the fitting analysis are discussed in detail in the following. First, we discuss the origin of Signal A. According to the previous studies of BCP/metal layered film samples, the formation of gap states have been reported at the BCP/metal interfaces by the interactions between metal and lowest unoccupied molecular orbital (LUMO) of BCP when the metal with a work function of ≤ 4.3 eV is used for layered films^42,43^. In the case of Al with a work function of 4.2 eV, a charge transfer has occurred between BCP and Al, which forms electric double layers due to the presence of gap states^42,43^. As a result, we have observed the ESR signal of BCP anions, and electrons can be injected into BCP from the Al metal without injection barriers^43^. From the observation of the motional narrowing of the ESR linewidth with a Lorentzian lineshape, we have concluded that a majority of the spins at the interfaces between BCP and Al are mobile^43^. In this study, we utilize the layered film of BCP/LiF/Al in the OLED structure. To investigate the effect of the insertion of LiF between BCP and Al, we fabricated a BCP/LiF/Al layered film and performed the ESR measurement. Solid line in Fig. 3a shows the ESR spectrum of the BCP/LiF/Al layered film; dashed line in Fig. 3a shows the fitting result of Signal A shown in Fig. 2a. The parameters of the observed ESR signal were *g* = 2.0031 ± 0.0001 and Δ*H*_pp_ = 0.35 ± 0.04 mT (Supplementary Table S1). The overall features of the signal such as *g*-factor correspond to that of Signal A (see Fig. 3a) within experimental errors; it also accords with those of the ESR signals of the BCP/Al layered film^43^. The low S/N ratio of the observed signal is ascribed to the low ESR intensity of the BCP/LiF/Al layered film. In addition, it has been reported that BCP films show no ESR signal^43^. Moreover, we confirm that LiF films show no ESR signal. Furthermore, the Lorentzian lineshape of Signal A is consistent with that of the layered films of BCP/Al and BCP/LiF/Al^43^. Therefore, we demonstrate that the origin of Signal A obtained from the fitting analysis is due to the gap states at the BCP/LiF/Al interface.

The origin of Signal B is discussed as follows. It has been reported that holes (positive polarons) in PEDOT:PSS are generated because hole doping happens in PEDOT by PSS and thus an ESR signal of PEDOT:PSS is observed^33,41^. The holes in PEDOT are mobile, which gives the ESR signal with a Lorentzian lineshape^33^. In this study, since we used the PEDOT:PSS film as the hole transport layer in the OLED, we performed the ESR measurement of a PEDOT:PSS film to confirm the contribution of PEDOT:PSS to the ESR signals of the OLED. As a result, the ESR parameters were obtained from the ESR spectrum of the PEDOT:PSS layer as *g* = 2.0028 ± 0.0001 and Δ*H*_pp_ = 0.28 ± 0.03 mT, as shown in solid line in Fig. 3b. We find that these parameter values (*g*-factor and Δ*H*_pp_) and peak-to-peak ESR intensity of the PEDOT:PSS film almost accord with those of Signal B (see dashed line in Figs. 3b and 2b). Therefore, the origin of Signal B is found to be holes in PEDOT:PSS in the OLED.

Next, we analyze the ESR spectra after the light emission (*V*_bias_ ≥ 4.5 V). Figure 3c shows the comparison between before (*V*_bias_ = 4.0 V) and after (*V*_bias_ = 4.5 V) the light emission. We find that the intensity of the spectrum at *V*_bias_ = 4.5 V is larger than that of the spectrum at *V*_bias_ = 4.0 V. Also, as shown in Fig. 3c, the lineshape of the signal at *V*_bias_ = 4.5 V is slightly different from that at *V*_bias_ = 4.0 V, with a component slightly shifted toward the high magnetic field side. In other words, simply increasing the intensity of the signal at *V*_bias_ = 4.0 V does not reproduce the signal at *V*_bias_ = 4.5 V. This means that another component is present in the signal at *V*_bias_ = 4.5 V. Figure 3d shows the difference spectrum between the two spectra. The ESR parameters of the difference spectrum were obtained as *g* = 2.0030 ± 0.0001 and Δ*H*_pp_ = 0.40 ± 0.04 mT. The ESR spectra at *V*_bias_ > 4.5 V were as large as that at *V*_bias_ = 4.5 V and we have observed the similar difference in ESR spectra at higher voltages such as 6.0 V and 4.0 V, as mentioned above (see Supplementary Fig. S3). Thus, we newly define this difference spectrum as Signal C, which are listed in Supplementary Table S1. Although the *g*-factor of Signal C appears to be close to those of Signal A and Signal B, the *g*-factor and Δ*H*_pp_ can be distinguished from those of Signal A and Signal B (see Fig. 2c and Supplementary Table S1). These results support the existence of Signal C. Figure 2c shows the result of the fitting analysis for the ESR spectrum (*V*_bias_ = 4.5 V) after the light emission. We can reproduce the ESR spectra after the light emission using 3 components, that is, Signal A due to the generating gap states at the BCP/LiF/Al interface, Signal B due to holes in the PEDOT:PSS film, and Signal C obtained from the differential spectrum between those at *V*_bias_ = 4.5 V and 4.0 V. Since Signal C is observed after the light emission and is different from those of the BCP/LiF/Al layered film (Signal A) and PEDOT:PSS film (Signal B), which are materials except for the light-emitting layer in the OLED, it is supposed that the origin of Signal C is related with spins of charges in the light-emitting layer. To further confirm the origin of Signal C, we performed an ESR study of a 3ACR-TRZ:CBP film used as the light-emitting layer. As a result, no ESR signal was observed at room temperature, which means that neutral molecules of 3ACR-TRZ and CBP do not have spins. Thus, Signal C can only be observed when charge doping is performed, as discussed below.

To investigate the origin of Signal C in detail, we performed density functional theory (DFT) calculation with Gaussian 09. The molecules for the calculation are 3ACR-TRZ and CBP used for the light-emitting layer. We calculated *g*-factors of the cationic and anionic states for the two molecules, respectively. Here, we performed the DFT calculation with structure optimization using a functional UB3LYP with a basis function 6-31+G(d,p). In the *g*-tensor calculation, we define the coordinate axes of *x*-, *y*-, and *z*-axis as shown in Supplementary Fig. S4. Since an anisotropy of the *g*-factor was not observed in the ESR measurements, the materials with observed spins are found to have no molecular orientation. Thus, we calculated an averaged *g*-factor (*g*_ave_) of the principal values (*g*_*x*_, *g*_*y*_, *g*_*z*_) of the *g*-tensor components as ${g}_{\mathrm{ave}}= \sqrt{\left({g}_{x}^{2}+{g}_{y}^{2}+{g}_{z}^{2}\right)/3}$. As a result, the *g*_ave_ of cationic 3ACR-TRZ and cationic CBP is determined as *g*_ave_ = 2.00289 and *g*_ave_ = 2.00297, and that of anionic 3ACR-TRZ and anionic CBP is determined as *g*_ave_ = 2.00275 and *g*_ave_ = 2.00258, respectively. The calculation results are summarized in Table 1. The *g*-factor (*g* = 2.0030) obtained from Fig. 3d is consistent with the *g*_ave_ of cationic CBP (*g*_ave_ = 2.00297). The *g*_ave_ values of both anionic states of 3ACR-TRZ and CBP are not consistent with the *g*_ave_ of cationic CBP (see Table 1). Therefore, the origin of Signal C is found to be due to long-lived or accumulated holes (cations) of CBP in the light-emitting layer after the hole transfer from the PEDOT:PSS layer to the light-emitting layer along with the start of the light emission. The spin density distributions for the cationic and anions states of 3ACR-TRZ and CBP were calculated by the DFT method as shown in Supplementary Fig. S4. Supplementary Fig. S4b,c show the spin density distribution of the cationic and anionic states of 3ACR-TRZ, respectively. We confirm that the spin density distribution of three acridan (ACR) units and one triazine (TRZ) unit is separated. Since the torsion angle between ACR and TRZ is nearly 90 degrees by steric hindrance, the localization and separation of spin density occur as known for highest occupied molecular orbital (HOMO) and LUMO of 3ACR-TRZ^21^. Supplementary Fig. S4e,f show the spin density distribution of the cationic and anionic states of CBP, respectively. Since CBP has a plane molecular structure, spins are delocalized and widely distributed in CBP.

**Table 1:** Calculated principal values of the *g*-tensor components and their averaged value (*g*_ave_) for 3ACR-TRZ and CBP molecules obtained from the DFT calculations.

Molecule and state	*g*_*x*_	*g*_*y*_	*g*_*z*_	*g*_ave_
3ACR-TRZ cation	2.00278	2.00276	2.00314	2.00289
3ACR-TRZ anion	2.00297	2.00347	2.00181	2.00275
CBP cation	2.00337	2.00281	2.00274	2.00297
CBP anion	2.00188	2.00248	2.00338	2.00258

### Relation between ESR and OLED characteristics

The relation between the ESR characteristics and luminance of the OLED is an interesting issue to investigate the effect of internal charge states in the OLED on the performance of the OLED. Figure 2d shows the *V*_bias_ dependence of *N*_spin_ derived from the ESR signal of the OLED and the luminance (*L*) observed during the ESR measurements. We find that *N*_spin_ decreases with the rise in *V*_bias_ before the light emission (*V*_bias_ = 0–4.0 V). The reason for the *N*_spin_ decrease will be discussed in detail later. In addition, we confirm that the *N*_spin_ rapidly increases at *V*_bias_ = 4.5 V in comparison to that at *V*_bias_ = 4.0 V. This *N*_spin_ increase seems to relate with Signal C as discussed above, which is partially ascribed to the formation of long-lived or accumulated cations of CBP with a lifetime of > 10 μs in the light-emitting layer after the light emission (*V*_bias_ ≥ 4.5 V). We comment that the *N*_spin_ number is given in absolute value in this study because in the device structure of this experiment, spins present in the PEDOT:PSS layer, light-emitting layer, and BCP/LiF/Al layer with different areas are observed, as shown in Fig. 1d, and the *N*_spin_ per unit area of the active layer cannot be defined.

Interestingly, we observe a gradual decrease in *N*_spin_ before the light emission with the rise in *V*_bias_ after device operation (0 < *V*_bias_ ≤ 4.0 V) in contrast to the case after the light emission (*V*_bias_ ≥ 4.5 V) as shown in Fig. 2d. There are two likely reasons for the *N*_spin_ decrease. One reason may be the recombination between holes (positive polarons) in the PEDOT:PSS layer and leakage electrons injected from the cathode passed through the light-emitting layer by *V*_bias_; we will discuss this first reason in more detail later. Another reason may be the formation of bipolarons by increasing the polaron density in the PEDOT:PSS layer^44–48^, where a bipolaron is the combination of two polarons and has no spin^44–48^. However, the latter reason will be ruled out later by the following results for OLEDs with MoO_3_. Also, the low hole density of PEDOT:PSS in the OLED before light emission seems to support this exclusion.

### Leakage electrons and the suppression in OLEDs with MoO_3_

**Figure 4:**
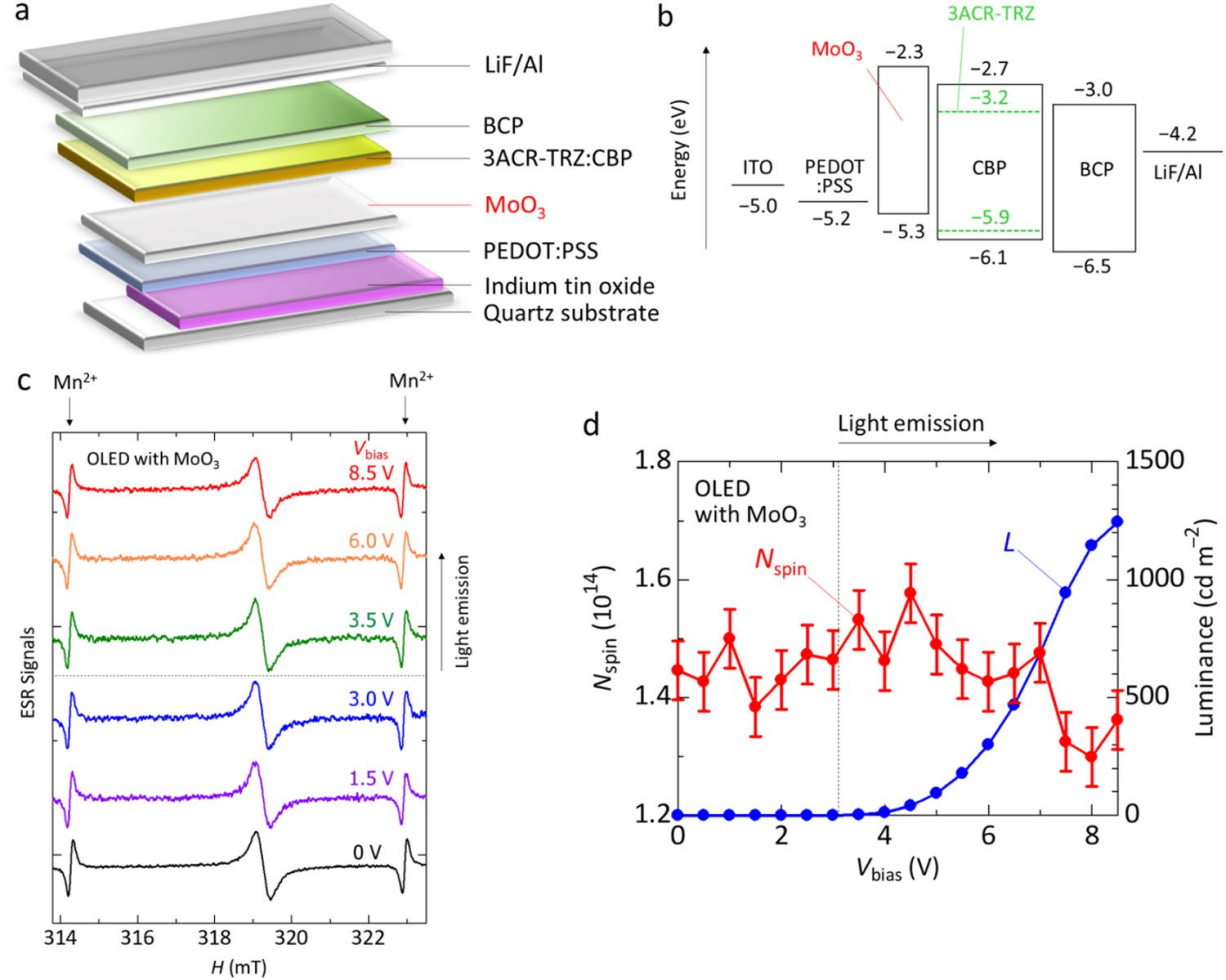
Device structure and operando ESR study of OLED with MoO_3_. (**a**) Schematic structure of the OLED with MoO_3_. (**b**) Energy level diagram of the OLED with MoO_3_. (**c**) *V*_bias_ dependence of the operando ESR spectra of the OLED with MoO_3_. (**d**) Dependence of *N*_spin_ derived from ESR spectra and the luminance (*L*) of the OLED with MoO_3_ on *V*_bias_ during device operation.

To clarify the reason for the *N*_spin_ decrease in the OLED before the light emission in detail, we performed an operando ESR measurements of the OLED with a further electron blocking layer MoO_3_; the experimental results are shown in Fig. 4. The actual composition of molybdenum oxide may be MoO_3−*x*_ where *x* represents the amount of oxygen defects but will be denoted MoO_3_ for simplicity. We fabricated the OLED in which we inserted a MoO_3_ layer between the PEDOT:PSS layer and light-emitting layer (Fig. 4a,b), and studied the *V*_bias_ dependence of the ESR spectra and *N*_spin_ (Fig. 4c,d). The component structure of OLEDs with the electron blocking layer MoO_3_ is ITO (150 nm)/PEDOT:PSS (35 nm)/MoO_3_ (7.5 nm)/3ACR-TRZ(16 wt%):CBP (55 nm)/BCP (30 nm)/LiF (1 nm)/Al (80 nm), which is the same structure mentioned in the above results and discussion except for the MoO_3_ insertion. The energy levels of the conduction band minimum (CBM) (− 2.3 eV) and valence band maximum (VBM) (− 5.3 eV) of MoO_3_ in Fig. 4b are taken from Refs.^31,49–51^, where MoO_3_ acts as a hole-transport layer connected with Ag hole collecting electrodes^31,49–51^. The energy level of the CBM of MoO_3_ (− 2.3 eV) is shallower than the LUMO levels of the light-emitting layer (Fig. 4b). Also, the CBM of MoO_3_ is thought to be shallower than the LUMO level of PEDOT:PSS (− 2.4 eV)^52^. Thus, MoO_3_ can play a role of a further electron blocking layer, which further prevents the leakage electron transfer from the light-emitting layer to the PEDOT:PSS layer. MoO_3_ was used because of its resistance to organic solvents. This layer serves as a substrate when spin-coating the light-emitting layer. If tris(4-carbazoyl-9-ylphenyl)amine (TCTA) or *N*,*N'*-diphenyl-*N*,*N'*-bis(1-naphthyl)-1,1'-biphenyl-4,4'-diamine (*α*-NPD or NPB) were used, they could dissolve during spin-coating of the light-emitting layer. MoO_3_ is a metal oxide and not an organic material. Therefore, the thickness of the MoO_3_ film is important, and in this study, a thickness of 7.5 nm, which is commonly used as a hole transport layer in organic solar cells^31,49^, was adopted. For the energy levels of MoO_3_, deeper energy levels of CBM (− 6.6 or − 6.7 eV) and VBM (− 9.6 or − 9.7 eV) than those in Fig. 4b have been reported, where the electron blocking ability of MoO_3_ with the very deep Fermi level is thought to be excellent^53,54^.

The electroluminescence characteristics of the OLEDs with MoO_3_ are as follows. When we fabricated devices using the conventional ITO substrate (25 mm × 25 mm), we obtained electroluminescence characteristics as shown in Supplementary Fig. S5. The luminance reached 19,145 cd m^−2^ at *V*_bias_ = 10.0 V. The maximum current efficiency was obtained as ~ 12.8 cd A^−1^ at *V*_bias_ = 7.8 V, which was evaluated from the data shown in Supplementary Fig. S5. The OLED emitted green light due to 3ACR-TRZ as observed for the OLEDs without MoO_3_ mentioned above. These characteristics are similar to those of the previous study^21^. When we fabricated devices for ESR measurements using the rectangular ITO substrate (3 mm × 25 mm, see Fig. 1d), the OLED emitted green light due to 3ACR-TRZ, which is like that observed for the OLEDs fabricated with the conventional ITO substrate (25 mm × 25 mm) mentioned above. The turn-on voltage was *V*_bias_ = 3.1 V, and the luminance reached 1245 cd m^−2^ at *V*_bias_ = 8.5 V. The maximum current efficiency was obtained as ~ 6.4 cd A^−1^ at *V*_bias_ = 7.5 V (Fig. 4d). The reason for the lower device performance obtained with the devices used for the ESR measurements may be due to the lower film quality compared to the conventional substrate used. Figure 4c shows the *V*_bias_ dependence of ESR spectra of the OLED with MoO_3_ before and after the light emission. These ESR spectra are not described by the single component as in the case of the OLED without MoO_3_, and thus we analyzed the ESR spectra before and after the light emission with the same fitting method used for the case of the OLED without MoO_3_. The fitting results are shown in Supplementary Fig. S6 and Supplementary Table S1. As in the case of the OLED without MoO_3_, we find that the ESR spectra are reproduced by the fitting curve with 2 components of Lorentzian (Signal A and Signal B) and 3 components of Lorentzian (Signal A, Signal B, and Signal C) before and after the light emission, respectively. Thus, the insertion of the MoO_3_ layer is found to have no large effect on the ESR signals. Figure 4d shows the *V*_bias_ dependence of *N*_spin_ evaluated from the observed ESR spectra. From Fig. 4d, we confirm that the decrease in *N*_spin_ before the light emission is suppressed in contrast to that without MoO_3_ shown in Fig. 2d. That is, by inserting the MoO_3_ layer, the leakage electron transfer from the light-emitting layer to the PEDOT:PSS layer is prevented, and the charge recombination between holes in the PEDOT:PSS layer and electrons leaked from the light-emitting layer is suppressed.

In addition, since the energy level of the VBM of MoO_3_ is located between the work function of the PEDOT:PSS layer and the HOMO levels of the light-emitting layer, it is thought that the hole injection from the PEDOT:PSS layer to the light-emitting layer smoothly occurs. That is, the charge recombination between holes and electrons in the light-emitting layer is expected to efficiently occur by inserting MoO_3_. In fact, the turn-on voltage of the OLED with MoO_3_ was *V*_bias_ = 3.1 V, which was 1.4 V less than that without MoO_3_. We newly find that MoO_3_ has a function of electron blocking layer in the OLED with the PEDOT:PSS hole transport layer, which performs a low-voltage drive. At *V*_bias_ > 3.1 V, we observe that the *N*_spin_ tends to increase by generating long-lived holes in CBP in the light-emitting layer similarly observed for Fig. 2d. Moreover, we observe an enhancement of the luminance for the OLED with MoO_3_ compared to that without MoO_3_ (see Figs. 4d and 2d). This luminance enhancement is reasonably ascribed to the electron blocking effect by the MoO_3_ layer mentioned above. It has been reported that performance of OLEDs is improved by optimizing the thickness of a hole-injection layer of MoO_3_ inserted between ITO and *α*-NPD (or NPB)^55^. For the device structure used in this study, optimization of the MoO_3_ film thickness may also provide further improvement of device characteristics, and it will be interesting to study the detailed MoO_3_ film-thickness dependence of ESR characteristics and device properties in the future. We comment the decrease in *N*_spin_ at *V*_bias_ ≥ 7.0 V, which may be caused by the charge recombination between holes in the PEDOT:PSS layer and electrons leaked from the light-emitting layer at higher *V*_bias_, as mentioned above.

## Conclusions

We fabricated the OLEDs with the TADF material 3ACR-TRZ and performed the operando ESR measurements to clarify the internal charge states in the OLEDs in detail. As a result, we have observed the variations of the ESR spectra of the OLEDs during the device operation. The experimental ESR spectrum of the OLED before the light emission was well reproduced by the two-component fitting (Signal A and Signal B). Signal A originated from the gap states at the BCP/LiF/Al interface, while Signal B from holes in the PEDOT:PSS layer. After the light emission, we have newly observed the ESR signal (Signal C) due to the long-lived or accumulated cations in CBP in the light-emitting layer. Moreover, by observing the *V*_bias_ dependence of the *N*_spin_ in the OLEDs, the decrease of *N*_spin_ has been confirmed.

To investigate the reason for the *N*_spin_ decrease in detail, we fabricated the OLEDs with MoO_3_ that is inserted between the PEDOT:PSS and light-emitting layers, and performed the operando ESR measurements. As a result, we have revealed that the charge recombination in the PEDOT:PSS layer with leakage electrons from the light-emitting layer causes the *N*_spin_ decrease and the high turn-on voltage of the light emission. In other words, the effect of the leakage electrons during driving the OLEDs has been verified at the molecular level by using the ESR method. In this study, no TADF-derived ESR signal was observed in the OLEDs using TADFs. This is different from the results of our previous OLEDs using the fluorescent material Alq_3_ (Ref.^28^). In that study, an Alq_3_-derived ESR signal was observed^28^. Therefore, these results may suggest that OLEDs with TADFs are superior to OLEDs with fluorescent materials in terms of suppressing charge formation and accumulation in light-emitting materials. The information obtained in this study will be useful for other materials and devices and be helpful to developments of OLED design with high efficiency and low driving voltage of the light emission. The leakage current affects not only TADF OLEDs but also fluorescent or phosphorescent OLEDs. Therefore, the findings of this study are important not only for TADF OLEDs but also for the development of fluorescent or phosphorescent OLEDs.

## Methods

### Fabrication of OLEDs

OLEDs for ESR measurements were fabricated using an ITO substrate with an active layer of 2.4 mm × 14 mm = 33.6 mm^2^. The ITO substrates were cleaned by ultrasonic cleaning with 2-propanole and acetone for 5 min and UV ozone cleaning for 30 min, respectively. After that, the PEDOT:PSS (Clevious AI4083) layer was spin-coated on the ITO substrate at 4000 rpm for 30 s and dried at 120 °C for 10 min. To form the light-emitting layer, 3ACR-TRZ was mixed with CBP and dissolved in 2 mL of chloroform. The mass ratio of 3ACR-TRZ and CBP was 1.6:8.4 and the combined weight was totally 10 mg. The dissolved solution was stirred with a stirring tip for more than 30 min. The light-emitting layer was then spin-coated at 1500 rpm for 30 s on the substrates and dried at 80 °C for 30 min. The electron transport layer (BCP), the electron injection layer (LiF), and the cathode (Al) were sequentially deposited by a vacuum deposition method at a pressure of 1.0 × 10^−5^ Pa. The deposition rate of BCP, LiF, and Al was 1.0–2.0 Å s^−1^, 0.1 Å s^−1^, and 5–20 Å s^−1^, respectively. In the case of the OLED inserting MoO_3_, the MoO_3_ layer was deposited between the PEDOT:PSS layer and the light-emitting layer. The thickness of MoO_3_ was 7.5 nm, and the deposition rate was 0.5 Å s^−1^ at a pressure of 1 × 10^−5^ Pa. The other layers were fabricated with the same method used for the OLEDs without MoO_3._

### ESR measurements

ESR measurements were performed with a rise of applied voltage bias (*V*_bias_) from 0 to 10 V with an increment of 0.5 V at room temperature. When the ESR measurements were performed, we integrated the ESR spectra at each *V*_bias_ for 15 times. The calibration of the *g*-factor was performed with a software program by a JEOL ESR system taking secondary high-order correction towards valid resonance magnetic field into account. The peak-to-peak ESR linewidth was evaluated by the difference between the two magnetic fields at a peak and valley of the ESR spectrum. The number of the spins (*N*_spin_) was calculated by the double integration of an ESR spectrum considering the absolute value of the *N*_spin_ of a standard Mn^2+^ marker sample that is calibrated with another standard sample of a solution (220 µL) of 4-Hydroxy-2,2,6,6-tetramethylpiperidine 1-oxyl (TEMPOL).

## Supplementary Information

Supplementary Information.

## Data Availability

The datasets used and/or analyzed during the current study available from the corresponding author on reasonable request.
