# Supplementary material for: Operando ESR observation in thermally activated delayed fluorescent organic light-emitting diodes
Source: Sci Rep. 2023 Jul 10;13:11109. doi: 10.1038/s41598-023-38063-3 (PMC10333204; doi:10.1038/s41598-023-38063-3)
Supplement: Supplementary file 1 — Supplementary Information. [file 41598_2023_38063_MOESM1_ESM.pdf]

Supplementary Information for

Operando ESR observation in thermally activated delayed fluorescent organic light-emitting diodes

Shintaro Yumoto[1], Junya Katsumata[1], Fumiya Osawa[1], Yoshimasa Wada[2], Katsuaki Suzuki[2], Hironori Kaji[2] and Kazuhiro Marumoto[1,3*]

[1]Department of Materials Science, Institute of Pure and Applied Sciences, University of Tsukuba, Tsukuba, Ibaraki 305-8573, Japan
[2]Institute for Chemical Research, Kyoto University, Uji, Kyoto 611-0011, Japan
[3]Tsukuba Research Center for Energy Materials Science (TREMS), University of Tsukuba, Ibaraki 305-8570, Japan

*Correspondence: marumoto@ims.tsukuba.ac.jp

This PDF file includes:

1. Electroluminescence characteristics of TADF OLEDs
2. Difference in ESR spectra of TADF OLEDs before and after light emission
3. ESR parameters of TADF OLEDs and layered thin films
4. Spin density distributions of 3ACR-TRZ and CBP by the DFT calculation.
5. Electroluminescence characteristics of TADF OLEDs with an electron blocking layer MoO_3
6. Operando ESR study of TADF OLEDs with an electron blocking layer MoO_3

1. Electroluminescence characteristics of TADF OLEDs

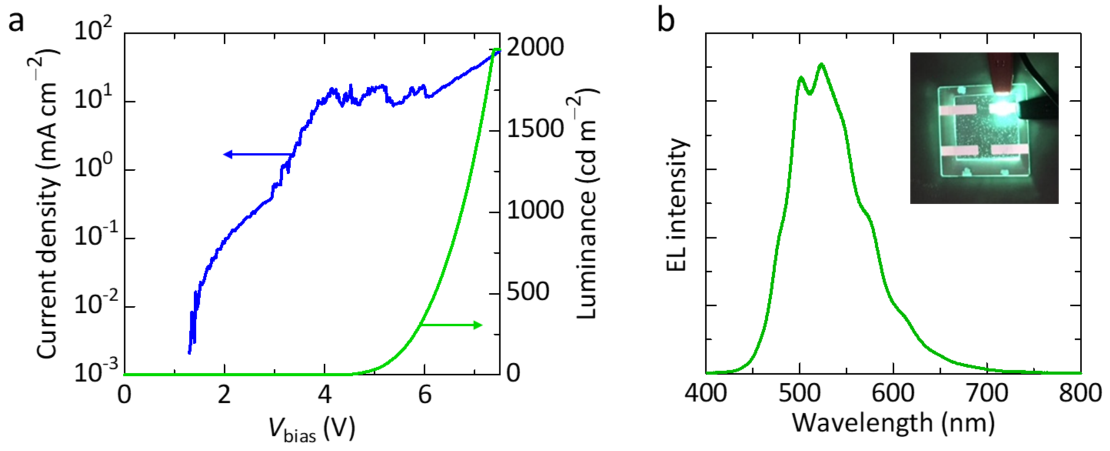

Supplementary Figure 1 | Electroluminescence characteristics of an OLED fabricated with a conventional ITO substrate. a, Dependence of current density and luminance characteristics of an OLED of ITO/PEDOT:PSS/3ACR-TRZ:CBP/BCP/LiF/Al on applied voltage (V_{bias}). **b**, Electroluminescence spectrum of the OLED. Inset shows a photograph of the green emission due to the TADF material 3ACR-TRZ in the OLED.

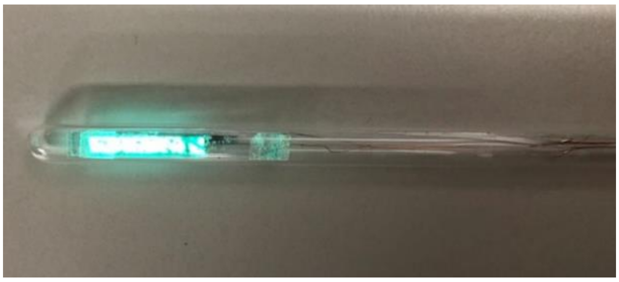

Supplementary Figure 2 | Electroluminescence characteristics of an OLED fabricated with a rectangular ITO substrate for ESR measurements. The photograph shows a green emission from the TADF material 3ACR-TRZ in an OLED device placed in a sample tube for ESR measurements.

2. Difference in ESR spectra of TADF OLED before and after light emission.

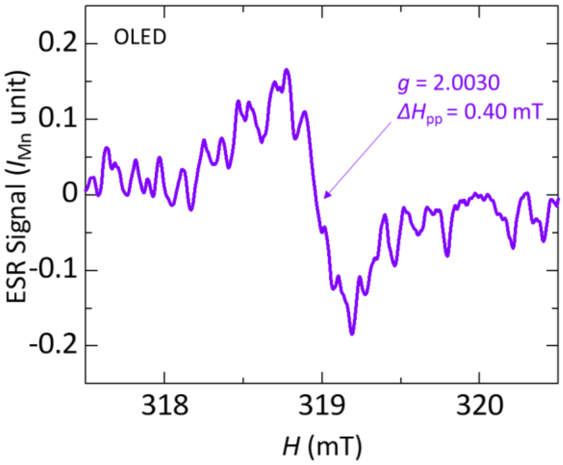

Supplementary Figure 3 | Difference in ESR spectra of a TADF OLED before and after light emission. Data is obtained by subtracting ESR spectrum at V_{bias} = 4.0 V from that at V_{bias} = 6.0 V for an OLED of ITO/PEDOT:PSS3ACR-TRZ:CBP/BCP/LiF/Al.

3. ESR parameters of OLEDs and layered thin films

Supplementary Table 1 | Summary of g-factor, peak-to-peak linewidth ΔH_{pp}, and type of lineshape for the ESR signals of OLEDs without and with MoO_3 and thin films.

	g factor	ΔH_{pp} (mT)	Lineshape
OLED (V_{bias} = 0 V)	2.0029 ± 0.0001	0.38 ± 0.02	2 components
OLED (V_{bias} = 4.5 V)	2.0029 ± 0.0001	0.40 ± 0.02	3 components
Fitting result (V_{bias} = 0 V) Signal A for OLED	2.0032 ± 0.0001	0.32 ± 0.04	Lorentzian
Fitting result (V_{bias} = 0 V) Signal B for OLED	2.0028 ± 0.0001	0.28 ± 0.04	Lorentzian
Fitting result (V_{bias} = 4.5 V) Signal A for OLED	2.0032 ± 0.0001	0.32 ± 0.04	Lorentzian
Fitting result (V_{bias} = 4.5 V) Signal B for OLED	2.0028 ± 0.0001	0.26 ± 0.04	Lorentzian
Fitting result (V_{bias} = 4.5 V) Signal C for OLED	2.0030 ± 0.0001	0.40 ± 0.04	Lorentzian
BCP/LiF/Al layered film	2.0031 ± 0.0001	0.35 ± 0.04	Lorentzian
PEDOT:PSS film	2.0028 ± 0.0001	0.28 ± 0.03	Lorentzian
Fitting result (V_{bias} = 0 V) Signal A for OLED with MoO_3	2.0032 ± 0.0001	0.28 ± 0.04	Lorentzian
Fitting result (V_{bias} = 0 V) Signal B for OLED with MoO_3	2.0028 ± 0.0001	0.29 ± 0.04	Lorentzian
Fitting result (V_{bias} = 3.5 V) Signal A for OLED with MoO_3	2.0032 ± 0.0001	0.28 ± 0.04	Lorentzian
Fitting result (V_{bias} = 3.5 V) Signal B for OLED with MoO_3	2.0028 ± 0.0001	0.31 ± 0.12	Lorentzian
Fitting result (V_{bias} = 3.5 V) Signal C for OLED with MoO_3	2.0030 ± 0.0001	0.35 ± 0.12	Lorentzian

4. Spin density distribution of 3ACR-TRZ and CBP

We calculated cationic states with UB3LYP/6-31G(d,p) and anionic states with UB3LYP/6-31G+(d,p) for 3ACR-TRZ and CBP as shown in Supplementary Figure 4. Supplementary Figure 4b,c shows the calculated spin density distribution of cationic (b) and anionic (c) states of 3ACR-TRZ, respectively. It is confirmed that the spin density distribution of three acridan (ACR) units and one triazine (TRZ) unit is well separated. Since the torsion angle between ACR and TRZ is nearly 90 degrees by steric hindrance, the spin localization and the separation occur as in the case of HOMO and LUMO of 3ACR-TRZ. Supplementary Figure 4e,f show the calculated spin density distribution of cationic (e) and anionic (f) states of CBP. Since CBP has the plane molecular structure, it has been confirmed that spins are delocalized and widely distributed in CBP.

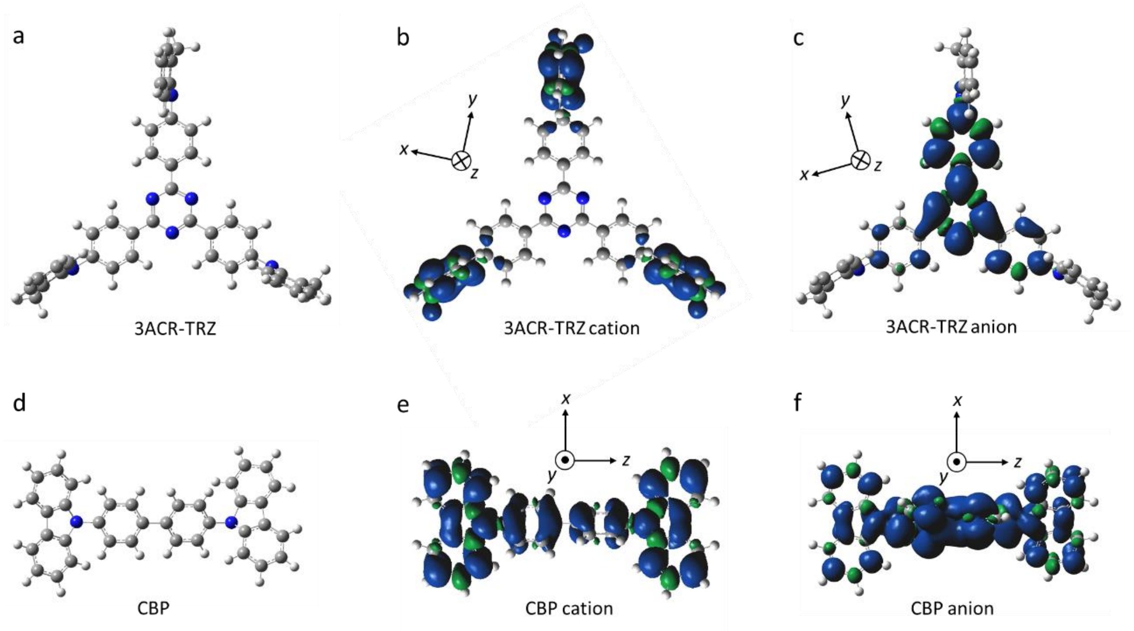

Supplementary Figure 4 | a, DFT-optimized molecular structure of 3ACR-TRZ. **b**, **c**, Spin density distributions of cation (**b**) and anion (**c**) of 3ACR-TRZ. **d**, DFT-optimized molecular structure of CBP. **e**, **f**, Spin density distributions of cation (**e**) and anion (**f**) of CBP.

5. Electroluminescence characteristics of TADF OLEDs with an electron blocking layer MoO_3

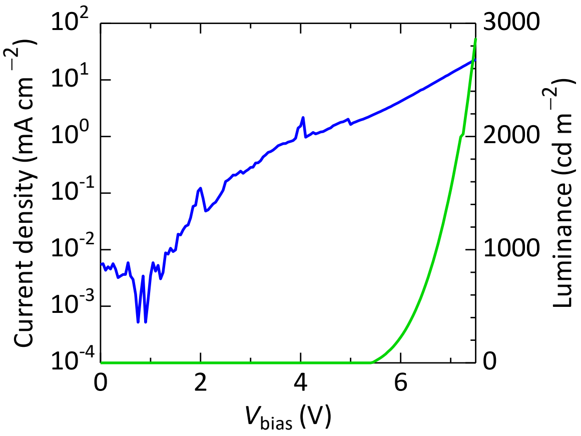

Supplementary Figure 5 | Electroluminescence characteristics of an OLED with an electron blocking layer MoO_3 fabricated with a conventional ITO substrate. Dependence of current density and luminance characteristics of an OLED of ITO/PEDOT:PSS/MoO_3/3ACR-TRZ:CBP/BCP/LiF/Al on applied voltage (V_{bias}).

6. Operando ESR study of TADF OLEDs with an electron blocking layer MoO_3

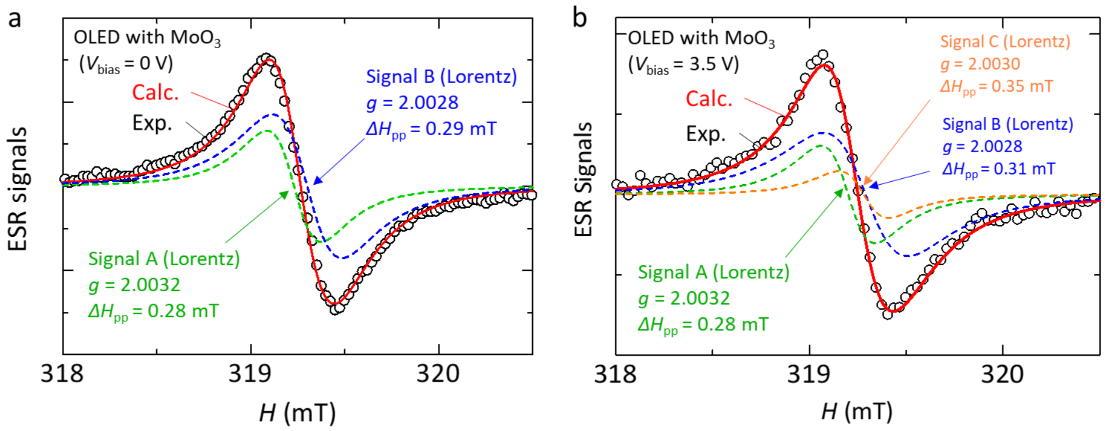

Supplementary Figure 6 | a, **b**, Fitting analyses of the ESR spectra of the OLED with an electron blocking layer MoO_3 before device operation at V_{bias} = 0 V (**a**) and after the light emission at V_{bias} = 3.5 V (**b**). The fitting results are summarized in Supplementary Table 1.
